# PPy@Fe_3_O_4_ nanoparticles inhibit the proliferation and metastasis of CRC *via* suppressing the NF-κB signaling pathway and promoting ferroptosis

**DOI:** 10.3389/fbioe.2022.1001994

**Published:** 2022-09-13

**Authors:** Zhilong Yu, Shanshi Tong, Chenyi Wang, Zizhen Wu, Yingjiang Ye, Shan Wang, Kewei Jiang

**Affiliations:** ^1^ Department of Gastroenterological Surgery, Laboratory of Surgical Oncology, Beijing Key Laboratory of Colorectal Cancer Diagnosis and Treatment Research, Peking University People’s Hospital, Beijing, China; ^2^ State Key Laboratory of Oncogenes and Related Genes, Shanghai Cancer Institute, Renji Hospital, School of Medicine, Shanghai Jiao Tong University, Shanghai, China

**Keywords:** colorectal cancer, nanoparticles, metastasis, NF-κB, ferroptosis

## Abstract

Colorectal cancer (CRC) is one of the most common cancers of the digestive tract, and patients with advanced-stage cancer have poor survival despite the use of multidrug conventional chemotherapy regimens. Intra-tumor heterogeneity of cancerous cells is the main obstacle in the way to effective cancer treatments. Therefore, we are looking for novel approaches to eliminate just cancer cells including nanoparticles (NPs). PPy@Fe_3_O_4_ NPs were successfully synthesized through a portable method. The characterization of transmission electron microscopy (TEM), Fourier-Transformed infrared spectrometer, and X-ray powder diffraction have further proved successful preparation of PPy@Fe_3_O_4_ NPs. NIR irradiation was used to test the photothermal properties of NPs and an infrared camera was used to record their temperature. The direct effects of PPy@Fe_3_O_4_ NPs on colorectal cancer cell DLD1 were assessed using CCK8, plate clone, transwell, flow cytometry, and western blotting in CRC cell. The effect of PPy@Fe_3_O_4_ NPs on neoplasm growth in nude mice was evaluated *in vivo*. This study demonstrated that PPy@ Fe_3_O_4_ NPs significantly inhibit the growth, migration, and invasion and promote ferroptosis to the untreated controls in colorectal cancer cells. Mechanical exploration revealed that PPy@Fe_3_O_4_ NPs inhibit the multiplication, migration, and invasion of CRC cells *in vitro* by modulating the NF-κB signaling pathway. Importantly, Ferroptosis inhibitors Fer-1 can reverse the changes in metastasis-associated proteins caused by NPs treatment. Collectively, our observations revealed that PPy@Fe_3_O_4_ NPs were blockers of tumor progression and metastasis in CRC. This study brought new insights into bioactive NPs, with application potential in curing CRC or other human disorders.

## Introduction

Colorectal cancer (CRC) ranks among the most common and devastating diseases of the digestive system globally (Bray et al., 2018; Siegel et al., 2021). There is no effective regime against this aggressive malignancy besides early surgical resection (Brenner et al., 2014). When patients are diagnosed with colorectal cancer, 15%–25% have liver metastases, and another 15%–25% develop them after radical resection of the primary tumor (Engstrand et al., 2018). However, radical resection of liver metastases is not possible in 80%–90% of cases (Modest et al., 2019). Among the reasons for this grim prognosis are the lack of obvious symptoms and reliable biomarkers for early diagnosis, as well as aggressive metastatic spread that leads to a poor response to treatment. Metastatic disease occurs in approximately 50% of diagnosed patients (Xu et al., 2019; Rebersek, 2020). Patients with advanced and metastatic cancer are generally treated with chemotherapy (Fan et al., 2021). The combination of radiation with chemotherapy is another option for treating unresectable, metastatic cancers (Koppe et al., 2005). Even so, both approaches are mainly aimed at improving survival rates and reducing symptoms of cancer (Aggarwal et al., 2013; Biller and Schrag, 2021).

With the rapid development of nanotechnology, nanoparticles (NPs) have provided a new approach for studying tumor therapies in recent years (Guan et al., 2022a; Zheng et al., 2021; Guan et al., 2022b). Nanomaterials refer to materials with at least one dimension ranging from 1 to 100 nm (Zheng et al., 2022). Due to their special dimensions, they have different optical, electromagnetic, biological, and thermal properties than general materials, making them more plastic (Sun et al., 2014; Enriquez-Navas et al., 2015; Duan et al., 2019; Li et al., 2021). The field has broad application prospects. Currently, nanomaterials treatment for cancer is mainly aimed at direct destruction of tumors, but in clinical treatment, high mortality rates of cancer are caused by the proliferation and metastasis of tumors, not the primary tumor site (Jiang et al., 2015; El-Toni et al., 2016). At present, the killing of tumors by nanoparticles mainly revolves around the photothermal properties and chemodynamic therapy of nanomaterials (Baek et al., 2016; Zhu et al., 2016; Tang et al., 2019), and nanoparticles’ direct effect on tumor cells has been little studied. Revealing the specific mechanism of nanoparticles’ effects on tumor cells is beneficial to promote the further application of nanoparticles in the human body.

Polypyrrole (PPy) is a kind of organic photothermal agent and photosensitizer, which can not only ablate cancer cells under infrared irradiation, and improve the effect of chemotherapy, but also has good biocompatibility, which can regulate cell adhesion, migration, protein secretion, and DNA synthesis as well as other processes under electric stimulation (Zhou et al., 2017; Liang et al., 2021; Miar et al., 2021). Human bodies require iron (Fe) as an essential trace element. Early studies found that the concentration of Fe in the body is negatively correlated with colorectal cancer. Therefore, people have high hopes for Fe treatment of tumors (Torti et al., 2018; Torti and Torti, 2020). There are also numerous studies that prove Fe supplementation can inhibit colorectal cancer development (Aksan et al., 2021; Phipps et al., 2021; Ploug et al., 2021). Whereas, some scholars believe that excess iron contributes to oxidative stress-induced colon damage and amplifies oncogenic signals. Therefore, the clinical application of Fe-containing drugs is limited (Padmanabhan et al., 2015; Wilson et al., 2018). It is possible to deliver nanoparticles to tumors through enhanced permeability and retention effect (EPR), and decompose iron ions directly in the tumor-specific microenvironment, which can avoid harming the normal colon.

**FIGURE 1:**
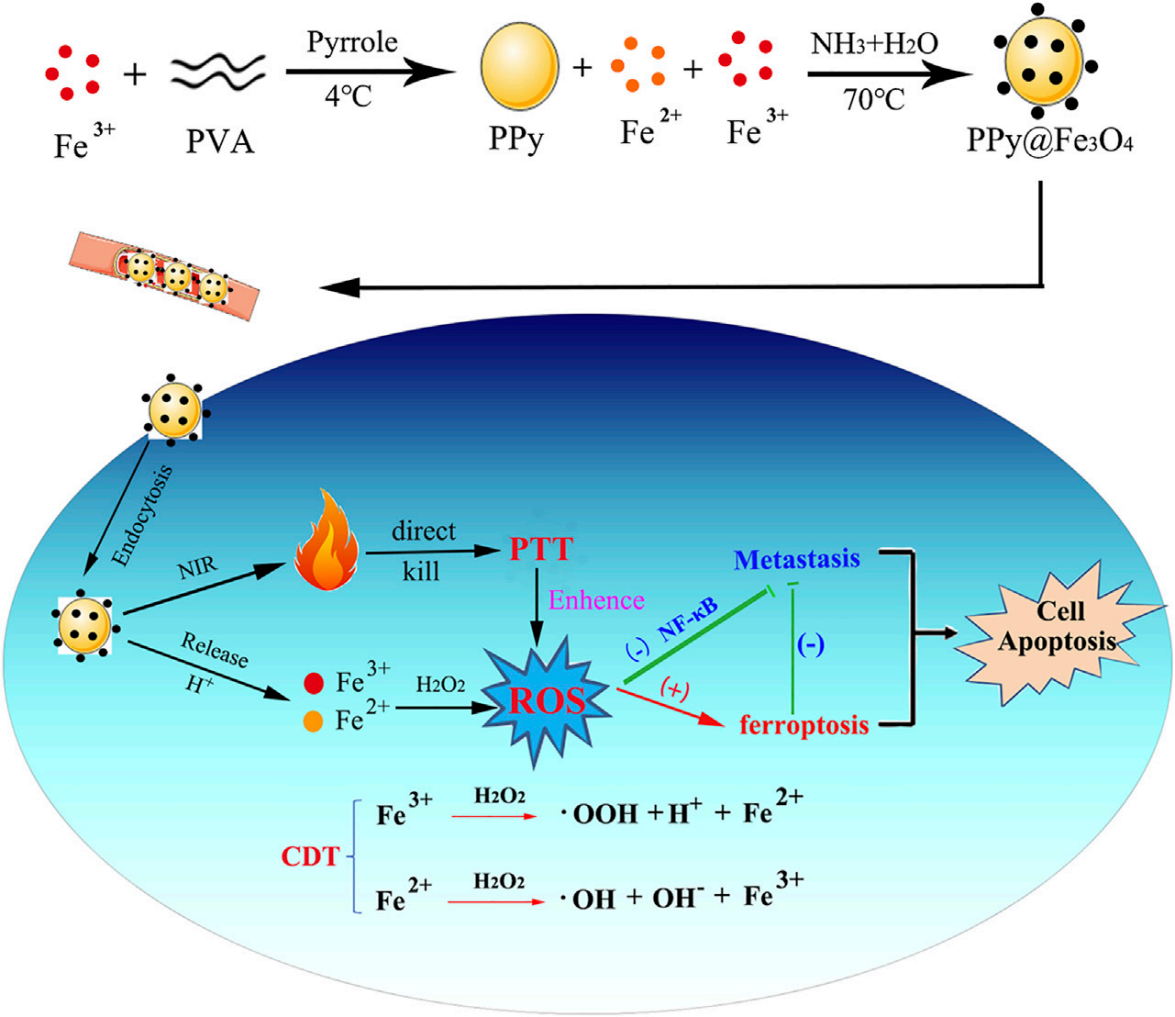
Scheme of the synthesis process and therapeutic mechanism of PPy@Fe_3_O_4_ NPs.

Our study design and manufacture a novel composite nanomaterial PPy@Fe_3_O_4_ and demonstrate that it can directly kill tumors through photothermal therapy (PPT) and chemodynamic therapy (CDT). As well as evaluating the basic properties and biosafety of PPy@ Fe_3_O_4_ NPs, we observed their effects on colorectal cancer cell proliferation, migration and invasion *in vivo* and *in vitro* (Figure 1).

## Materials and methods

### Synthesis of PPy@Fe_3_O_4_ NPs

Dissolve 0.75 g of polyvinyl alcohol (PVA) in 10 ml of deionized water. Heated to 95°C, after dissolving PVA, 0.373g ferric chloride powder (FeCl_3_ 2.30 mmol) was added to the above solution and stirred magnetically for an hour. Then the mixed solution was kept at 4°C and 69.2 μl pyrrole monomer (0.9970 mmol) was added slowly. After 4 h of stirring, the mixture was poured into a bowl. A dark green solution was produced, which indicated the successful synthesis of polypyrrole NPs. Then, 2.5 ml of the reaction solution was directly removed from the above steps, and then 15 ml of deionized water and 2 ml of ethanol were evenly mixed. Under the condition of full agitation, the temperature was rapidly heated to 70°C, and 1 ml of 1.0wt% ammonia solution was immediately dropped. After 30 min, inject another 1 ml 1.0wt% ammonia solution and keep the mixture at the same temperature for another 30 min. Centrifugation by separation (11,000 RPM; 50 min) PPy@Fe_3_O_4_ nanoparticles were collected and centrifuged (11,000 RPM; 50 min), washed three times with deionized water to remove impurities, and collected and dispersed in deionized water.

### Characterization of PPy@ Fe_3_O_4_ NPs and photothermal effect evaluation

The morphologies of NPs were evaluated via transmission electron microscopy (TEM). In order to determine the characteristics of NPs and their crystal structures, Fourier-Transformed Infrared (FTIR) spectrometers and X-ray powder diffraction methods were used. We then irradiated PPy@ Fe3O4 NPs with NIR lasers at different wavelengths (100, 200, and 400 μg/ml) at different concentrations. A thermal imaging camera was used to monitor and record the temperature changes of the solution during the heating and cooling process to calculate the photothermal conversion efficiency (η).

### Culture of the cancer cell lines

DLD1-1, SW480, and FHC colorectal cancer cell lines were purchased from ATCC. DLD1 and SW480 were colorectal cancer cells, and FHC was a normal colorectal epithelial cell. All cell lines were cultured in Dulbecco’s Modified Eagle’s Medium (DMEM) (Gibco, United States). All media were supplemented with 10% fetal bovine serum (FBS) and cells were grown in an incubator at 37°C and supplied with 5% CO_2_.

### Biosafety and flow cytometry analysis

In advance, DLD1, SW480, and FHC cells were plated in 96-well plates at 1*10^4^ cells per well and cultured for 24 h at 37°C under 5% CO_2_. At various concentrations, PPy@Fe_3_O_4_ was added to the culture media for 24 h, followed by 18 h of incubation. In accordance with the manufacturer’s instructions, relative cell viability was assessed using the Cell Counting Kit-8 (CCK-8, Yeasen, China).

### Transwell migration assay

Transwell migration assays were conducted in Corning-Costar migration chambers with a pore size of 8 mm for studying CRC cell migration in transfected suspensions. As soon as possible, transfected cells were seeded into an FBS-free medium and conditioned DMEM containing 10% FBS was poured into the lower chamber. In the following 48 h, we removed the cells on the upper membrane surface and fixed and stained the cells on the bottom membrane surface with methanol and crystal violet. We photographed cells from five random fields (×40 magnifications) under the light microscope.

### Western blotting

Equal amounts of samples were separated by 10% SDS-PAGE and transferred to PVDF membranes. Blocking membranes with 5% non-fat milk in TBST for 1 h, primary antibodies were incubated overnight at 4°C, followed by secondary antibodies at room temperature for 90 min. The immunoreactive bands were visualized using a ChemiLucent ECL kit (Millipore) and the ImageJ program (National Institutes of Health).

### Determination of intracellular ROS

In accordance with the manufacturer’s instructions, chloro-dihydrofluorescein diacetate (DCFH-DA) was used to determine intracellular ROS. Briefly, DLD1 cells were incubated with NPs (200 µg/ml^−1^) at pH 6.5 for 3.5 h, followed by 30 min of incubation with H_2_O_2_ (100 mM, 200 µl). The cells were placed on an ice box at 4°C. Then the medium was replaced by 1 ml DCFH-DA (10 µM).

### Animal experiments

All experiments on animals were conducted in accordance with “China National Standards for the Care and Use of Laboratory Animals” and were approved by the Ethics Committee of Renji Hospital Affiliated with Shanghai Jiaotong University School of Medicine. In order to establish colorectal cancer xenograft model, 20 male BALB/c athymic nude mice (4 weeks old) were randomly divided into four groups (*n* = 5) and injected subcutaneously with 1.0*10^7^ stable colorectal cells DLD1. A variety of intravenous preparations were administered: Control (groups 1), NIR(groups 2), NPs (200 µg/ml^−1^) (groups 3), NIR + NPs (groups 4). We used an 808 nm laser (1.0 W cm^−2^) to irradiate Groups 2 and 4 for 10 min respectively after 8 h and monitored temperature change by a thermal imaging camera. Prior to the mice being killed, tumor growth was monitored and measured with micrometer calipers every other day. After 28 days of treatment, immediately after harvest, organs and tumors were preserved in paraformaldehyde for further IHC testing and hematoxylin and eosin staining (H&E-stained).

### Statistics

All data are presented as mean ± SD. Statistical analyses were performed with the χ2 test or the Student's t-test (two-tailed unpaired). All the data were analyzed using Origin and Graphpad. Moreover, *p* < 0.05 is considered statistically significant.

## Results

### Construction and physical characterization of PPy@Fe_3_O_4_

**FIGURE 2:**
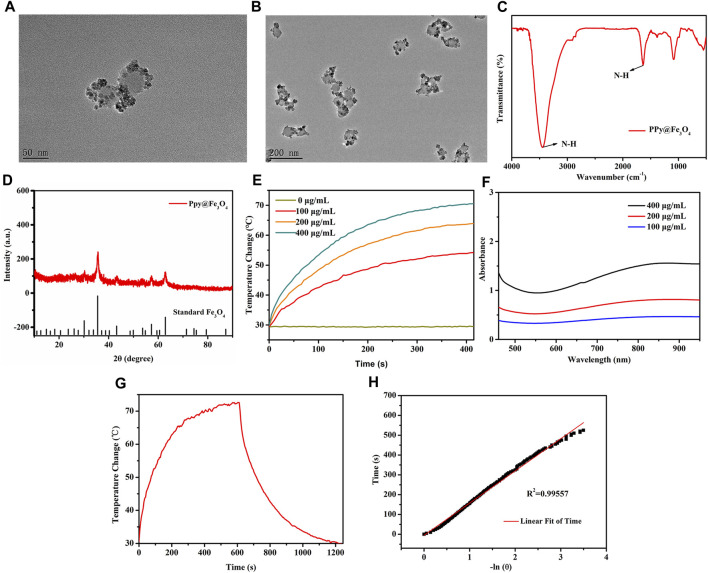
The characterization and photothermal properties of the PPy@Fe_3_O_4_ NPs. **(A,B)** High and Low TEM images of PPy@Fe_3_O_4_ NPs; **(C)** FTIR spectra of PPy@Fe_3_O_4_ NPs; **(D)** XRD spectra of PPy@Fe_3_O_4_ NPs; **(E)** UV-Vis-NIR absorption spectra of PPy@Fe_3_O_4_ NPs at different concentrations; **(F)** Temperature change curve with various concentrations of NPs; **(G)** Temperature curve of rising with irradiation and naturally cooling; **(H)** Linear regression curve of cooling process (red).

The PPy nanoparticles were firstly prepared, followed by ammonia addition at 70°C to convert Fe ions into Fe_3_O_4_ crystals. The Fe_3_O_4_ crystals were dispersed on the surface of PPy nanoparticles, forming PPy@Fe_3_O_4_ NPs with a size of ∼70 nm, as shown in Figures 2A,B. Each PPy nanoparticle incorporated many Fe_3_O_4_ crystals. The FTIR spectrum confirmed the successful formation of PPy by showing the characteristic absorption peaks (Figure 2C). Fe_3_O_4_ crystal structures were confirmed by X-ray diffractograms (XRD) of NPs (Figure 2D). These results illustrated that the PPy@Fe_3_O_4_ NPs have been successfully synthesized.

Since PPy was introduced to Fe_3_O_4_ NPs, they demonstrated a strong and broad absorption spectrum from the visible to near-infrared (Figure 2E). As the NPs concentration increase, the temperature also increases gradually under NIR irradiation (Figure 2F). Based on the temperature changes of the solution during heating/cooling process, we determined the photothermal conversion efficiency (ŋ value) of NPs (Figures 2G,H). The ŋ value was significantly higher than that of traditional PPT agents at 52%. The above results showed that the PPy@Fe_3_O_4_ NPs have excellent photothermal effects, which endowed good performance for PTT.

### PPy@Fe_3_O_4_ NPs inhibited growth and produced ROS *in vitro*

**FIGURE 3:**
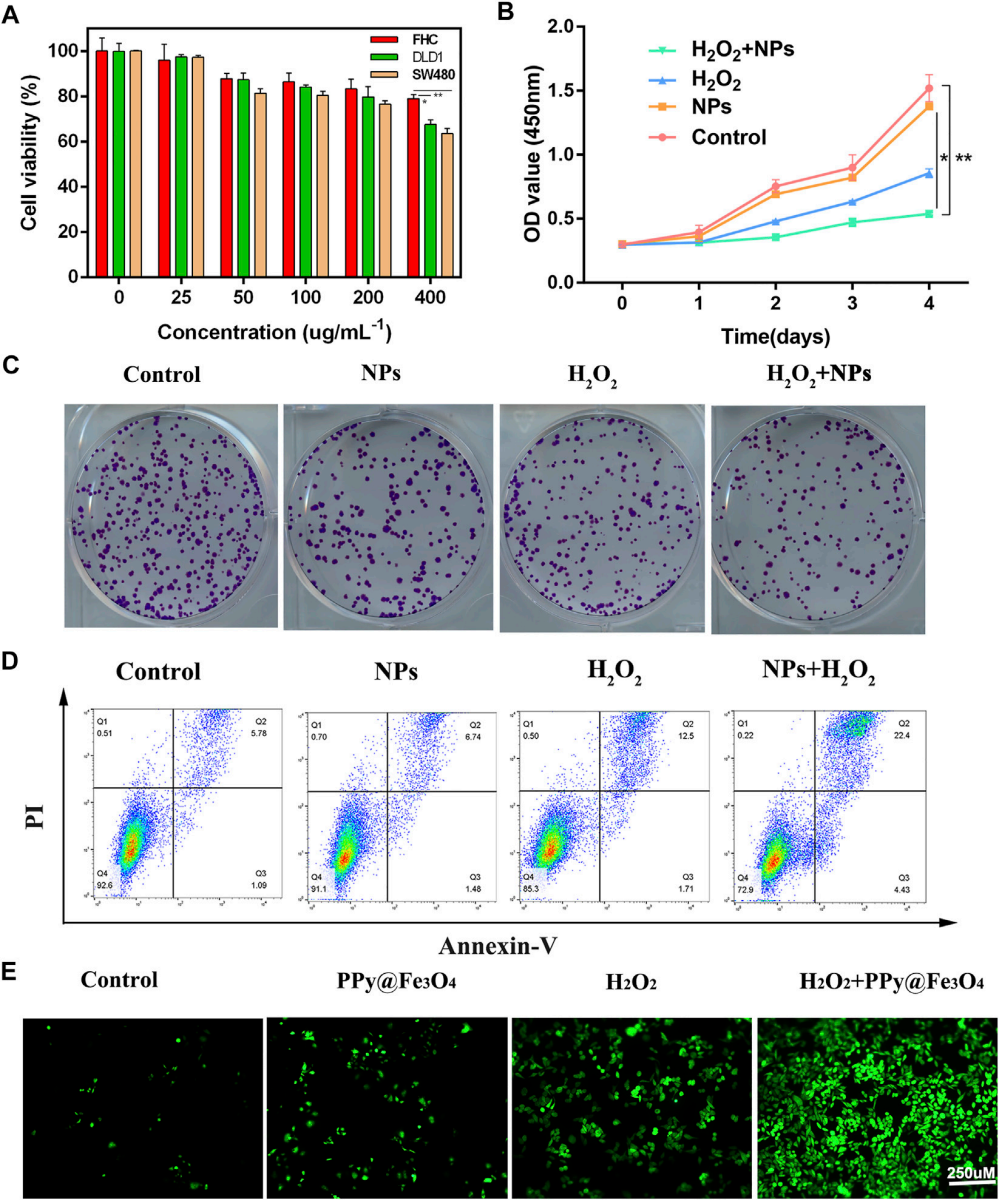
Effects of PPy@Fe_3_O_4_ NPs on regulating colorectal cancer cell growth, clone formation apoptosis and ROS generation. **(A)** Cell viability of the DLD1, SW480 and FHC cells after co-culture with NPs at different concentrations. **(B)** Cell viability CCK-8 assay in different groups. **(C)** Colony formation assay. Duplicated cells were subjected to the tumour cell colony formation assay in different groups. **(D)** Flow cytometric apoptosis assay. Colorectal cancer cell lines DLD1 were treated with H_2_O_2_, NPs, NPs + H_2_O_2_ or control, respectively, and then subjected to flow cytometric analysis. **(E)** Fluorescence images of DLD1 cells with various groups (Control, H_2_O_2_, NPs and NPs + H_2_O_2_). Scale bar: 250 µm **p < 0.01.

Biological applications of nanoparticles depend on their good biocompatibility. To evaluate its cytotoxicity, we used standard CCK-8 methods in DLD1, SW480, and FHC cells. As shown in Figure 3A, NPs exhibited excellent biocompatibility, except for NPs (400 ug/ml^−1^), with mildly stronger cytotoxicity due to their chemodynamic reactions. To simulate the tumor microenvironment *in vitro*, we added the appropriate amount of hydrogen peroxide during cell treatment. Therefore, colorectal cancer cells were divided into 4 groups: 1) Control, (b)H_2_O_2_, (C) NPs, (d)NPs + H_2_O_2_. DLD1 cells proliferation was significantly decreased by treatments with NPs and H_2_O_2_ in plating colony and CCK8 assays demonstrating that PPy@Fe_3_O_4_ functions biologically in colorectal cancer (Figures 3B,C). For cell apoptosis assay, NPs and H_2_O_2_ treated group promoted apoptosis in DLD1 cells (Figure 3D). To verify the ROS production of NPs in DLD1, we observe dichloro-dihydrofluorescein diacetate staining (DCFH-DA) under confocal microscopic conditions, ROS levels were significantly augmented in cells treated with NPs and H_2_O_2_, indicating a promoting effect on ROS generation (Figure 3E).

### PPy@Fe_3_O_4_ NPs suppressed metastasis and promoted ferroptosis in CRC cells

**FIGURE 4:**
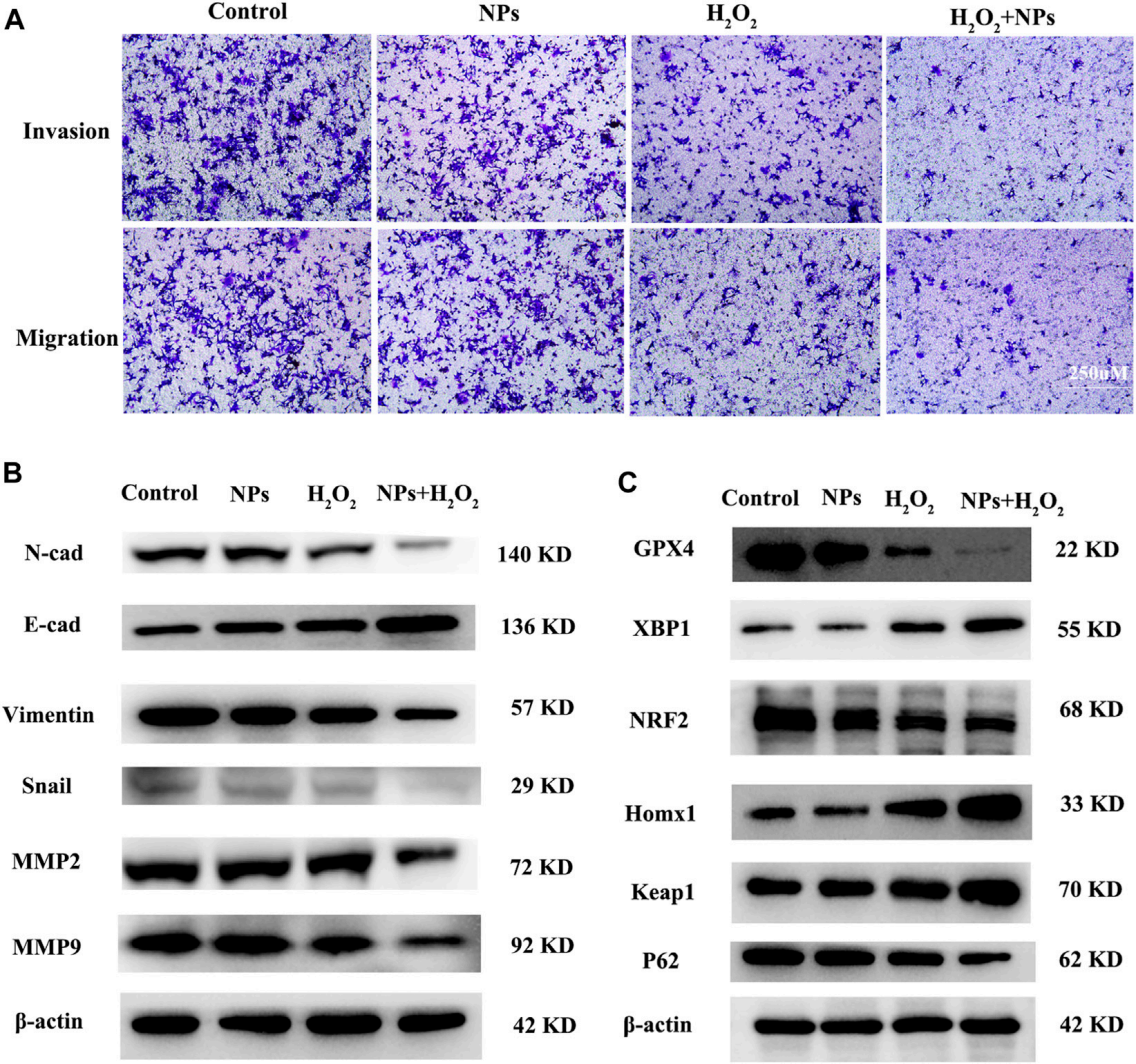
PPy@Fe_3_O_4_ NPs suppress cell migration and invasion, and promote cell ferroptosis *in vitro*. **(A)** Transwell migration and invasion assays of DLD1 cell with different treatment groups. **(B)** WB assays showed that metastasis-related proteins (E-Cadherin, N-Cadherin,Snail, MMP2, MMP9 and Vimentin) expression changed in different groups. **(C)** Ferroptosis-related proteins (GPX4, XBP1, NRF2, HOMX1 and Keap1) expression changed in the control group and other treated groups.

Transwell migration and invasion assays indicated that NPs and H_2_O_2_ treated group decreased the ability of migration and invasion (Figure 4A). Since epithelial-mesenchymal transition (EMT) plays a vital role in tumorigenesis, the relationship between NPs and EMT in CRC cells warranted further investigation. EMT biomarkers were used to identify whether NPs treated in CRC were related to EMT. The WB results showed that NPs inhibited the expression of the mesenchymal markers N-cadherin, Vimentin, Snail, MMP2, and MMP9, but induced the expressions of the epithelial marker E-cadherin (Figure 4B). Therefore, we inferred that PPy@Fe_3_O_4_ NPs inhibited tumor metastasis through inhibiting the EMT process.

In addition, studies also shown that the role of ROS in tumor cells is closely related to ferroptosis (Su et al., 2019; Chen et al., 2021a), and PPy@ Fe_3_O_4_ nanomaterials not only generate ROS in tumors, but also the constant conversion of Fe^2+^ and Fe^3+^ through the Fenton reaction, which also affects the iron ions metabolism. We speculated that NPs are associated with ferroptosis in tumor cells. Our data showed that PPy@Fe_3_O_4_ induced the expression of Xbp1, Homx1, and Keap1, but inhibited the expressions of GPX4 and NRF2 (Figure 4C). In addition, hydrogen peroxide has been reported to induce ferroptosis, which is consistent with our findings. Therefore, we inferred that PPy@Fe_3_O_4_ NPs can promote ferroptosis in CRC cells.

### PPy@Fe_3_O_4_ NPs inhibited EMT via the NF-κB signaling pathway

**FIGURE 5:**
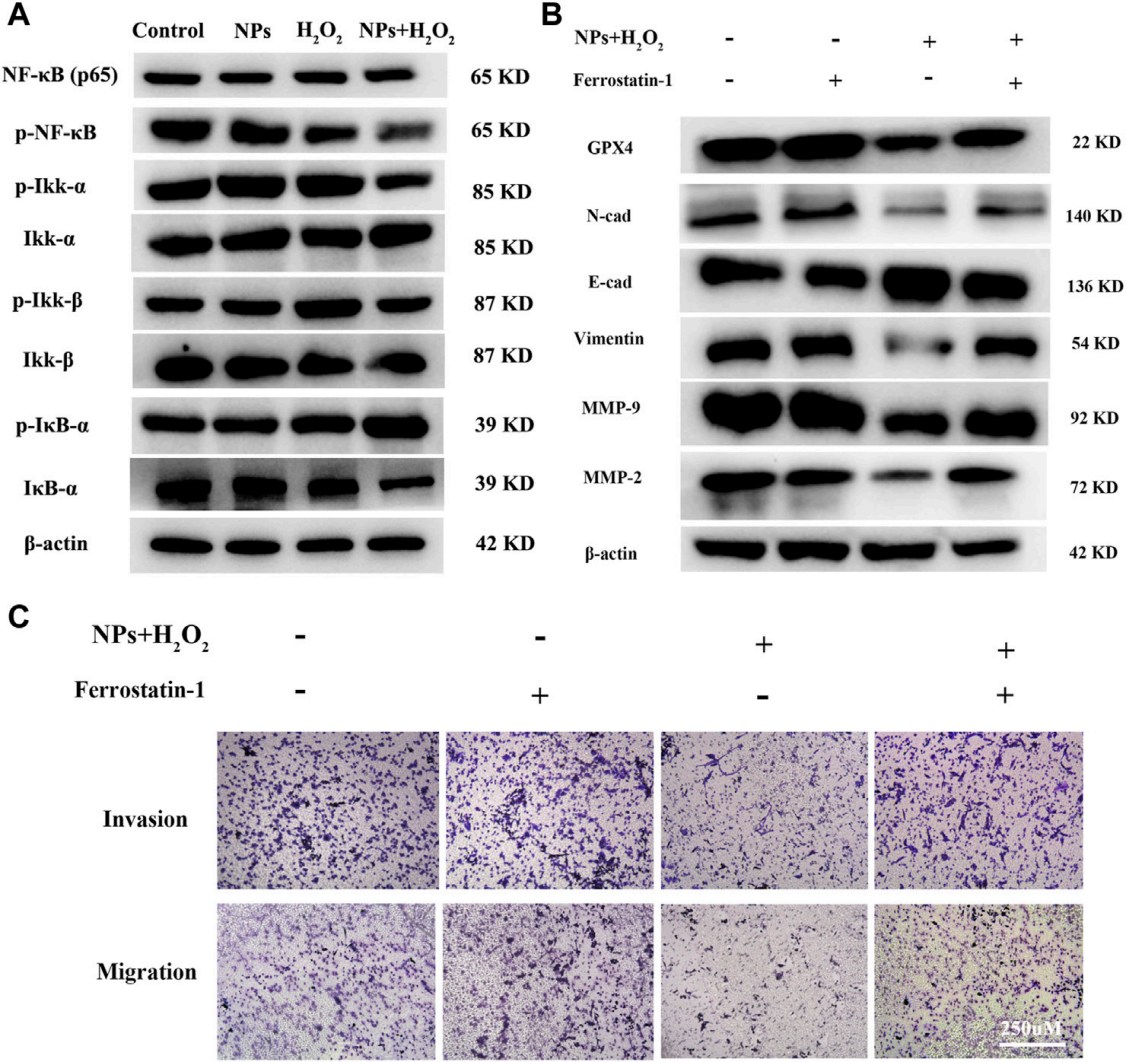
PPy@Fe_3_O_4_ NPs suppress CRC cells metastasis by promoting cell ferroptosis and inhibiting NF-κB signaling pathway. **(A)** Western blot. Colorectal cancer cell line DLD1 was treated with various groups (Control, H_2_O_2_, NPs and NPs + H_2_O_2_), and then subjected to Western blot analysis of the key proteins of the NF-KB signaling pathway (Ikk-β, p-Ikk-β, ikk-α, p-Ikk-α, NF-κβ, p-NF-κβ, IκB-α and p-IκB-α). **(B)** Effects of the ferroptosis inhibitor Ferrostatin-1 on PPy@Fe_3_O_4_ NPs-induced metastasis-related proteins expression. **(C)** Transwell showed that PPy@Fe_3_O_4_ NPs-induced cell migration and invasion were abolished after addition of the ferroptosis inhibitor Ferrostatin-1 in CRC cell.

NF-κB is involved in many cancer-related processes, including cell proliferation, apoptosis, angiogenesis, and metastasis in colorectal cancer (Vaiopoulos et al., 2013; Patel et al., 2018). A previous study reported excessive ROS can reduce NF-κB activation by inhibiting IκB protein degradation (Morgan and Liu, 2011). So we hypothesized whether PPy@Fe_3_O_4_ inhibits tumor cell metastasis by inhibiting NF-κB signaling. As part of this study, we measured the expression and activity of NF-κB in CRC cells treated with NPs. There was a decrease in the levels of p-IKKα and p-IKKβ in DLD1, as well as an increase in the amounts of p-IκBα after treatment with NPs and H_2_O_2_. P65 levels did not change significantly, but phospho-p65 expression decreased. We discovered that the expression of phosphorylated (p)p65, p-IKKα, p-IKKβ, and IκBα, which are essential for activating the NF-κB signaling pathway, were downregulated by NPs with H_2_O_2_ in DLD1 cells (Figure 5A).

Some studies have reported that there is an interaction between EMT and ferroptosis (Chen et al., 2020; Guan D. et al., 2022). GPX4, which is a negative regulator of ferroptosis, knockdown can enhance tumor cell oncogenic and metastatic activity (Huang et al., 2022). We suppose ferroptosis was increased after PPy@Fe_3_O_4_ treatment, and the metastatic ability of colorectal cancer cells was inhibited by increased GPX4 expression. After inhibition of tumor cell ferroptosis with ferroptosis inhibitors Ferrostatin-1 (Fer-1), western-blot analysis and transwell assays revealed increased metastatic potential of colorectal cancer cells, and the expression of EMT-related proteins was distinctly altered, with N-cadherin, Vimentin, Snail, MMP2 and MMP9 upregulated and E-cadherin downregulated (Figures 5B,C). These results demonstrated that PPy@Fe_3_O_4_ NPs inhibit CRC cells’ metastasis by promoting cell ferroptosis and inhibiting the NF-κB signaling pathway.

### *In vitro* cell experiment

**FIGURE 6:**
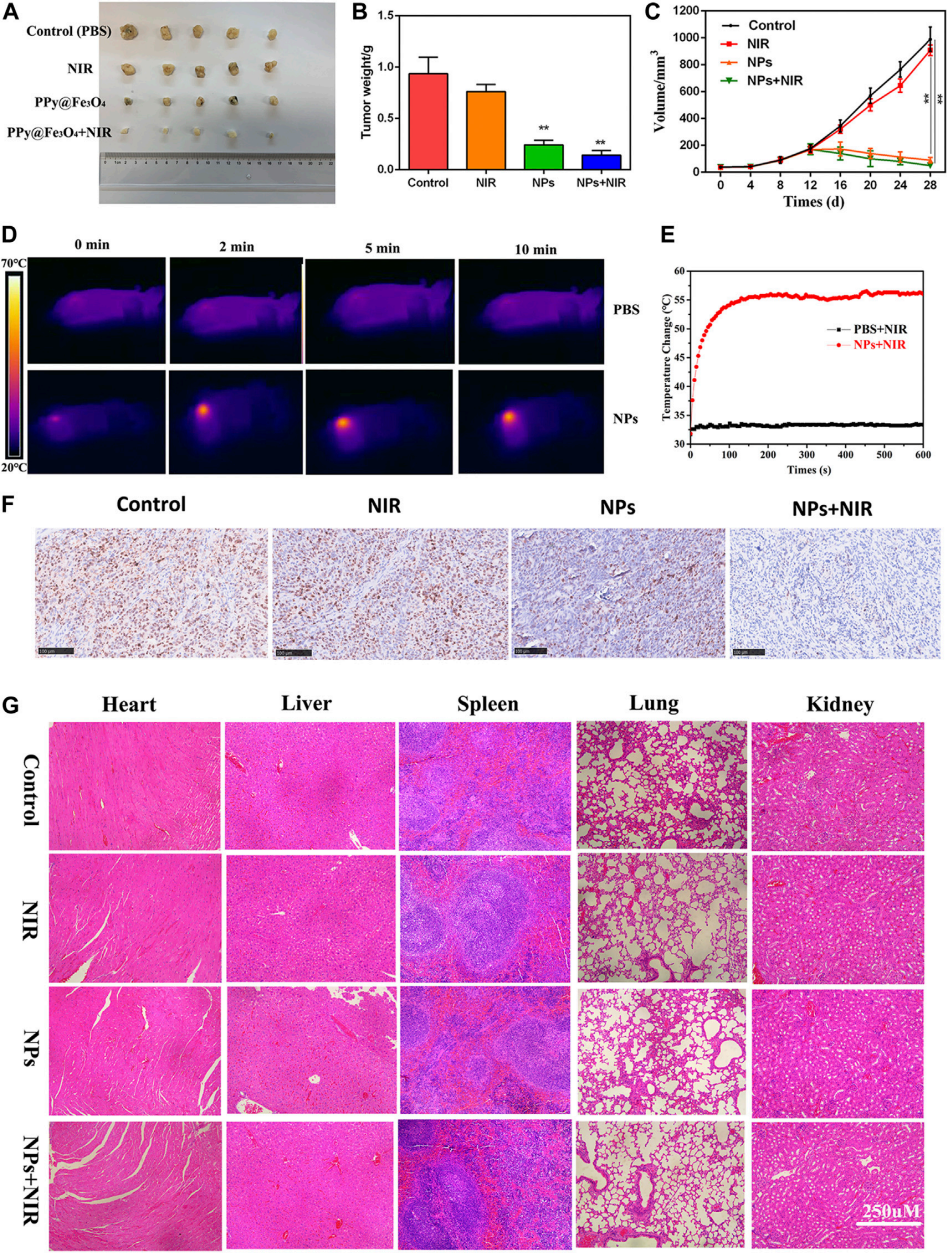
Anti-tumour activity of PPy@ Fe_3_O_4_ NPs in nude mouse tumour cell xenografts. **(A)** Images of subcutaneous xenograft tumors of DLD1 cells. **(B)** The final tumor weight of DLD1 cells was shown. **(C)** The tumor volume and change of different groups. **(D)** The temperature change and **(E)** infrared thermal imaging of the mice injected with PBS, NPs under laser irradiation. **(F)** Ki67 staining of the tumors in the control group and other treated groups. (scale bar: 100 μm). **(G)** H&E staining of the main organs from the control and treatment groups. (scale bar: 250 μm). **p < 0.01.

In order to investigate the roles of PPy@Fe_3_O_4_ NPs *in vivo*, a nude mouse xenograft model of colorectal cancer was constructed. Tumor volume was monitored every other day throughout the experiment. As a result of NPs treatment, tumor growth was significantly inhibited (Figures 6A–C). There was no difference in tumor growth between the NIR and control groups, demonstrating that NIR alone cannot inhibit tumor growth. However, due to the synergistic effects of PTT and CDT, the tumor growth in the NPs + NIR group was significantly inhibited. NIR group mice were irradiated with an 808 nm laser while their infrared thermal image and temperature were recorded simultaneously. Laser irradiation rapidly increased the temperature of the tumor in the NPs group to 55°C. It has been reported that apoptosis and necrosis of cancer cells can be induced when the temperature around the tumor is above 42°C (Sun et al., 2019). In contrast, the control group only experienced a very weak rise, less than 35°C (Figures 6D,E). In the colorectal tumor model, Ki-67, a marker of cell proliferation, was significantly downregulated in NPs + NIR groups after IHC analysis (Figure 6F). These results explicitly demonstrated that NPs with NIR could effectively prevent tumor growth *in vivo*. H&E staining of various treatment groups was carried out for the purpose of assessing the biosafety of NPs. According to the data, neither the control group nor other treatment groups showed obvious organ damage (Figure 6G), which further validated the PPy@Fe_3_O_4_ NPs were safe.

## Discussion

PPy@Fe_3_O_4_ NPs were successfully synthesized by an facile method. They exhibited an excellent photothermal effect and could produce abundant ROS for CDT in the tumor microenvironment. Furthermore, NPs are adequate to modulate cellular response on their own (Setyawati and Leong, 2017; Cen et al., 2021). First, we used CCK8 to detect the viability of normal cells and tumor cells exposed to different concentrations of NPs to judge the biosafety of NPs. We then demonstrated *in vitro* that NPs can restrain the accretion and metastasis of CRC cells and promote ferroptosis. Finally, we found that NPs inhibit CRC cells growth by inducing ferroptosis and inhibiting NF-κB pathway. *In vivo* experiment results further confirmed the inhibition of NPs on tumor growth.

Our team has long been committed to the practical application of photothermal technology. PTT and CDT of nanoparticles have enormous potential in cancer treatment (Huang et al., 2019; Zheng et al., 2021). CDT/PTT has demonstrated to be highly effective and relatively safe, and it can directly ablate cancer cells as well. Additionally, photothermal effects during PTT can speed up the Fenton-based process’ reaction rate and enhance CDT (Yu et al., 2021; Zhang et al., 2022). With the development of nanotechnology, various targeted and multifunctional nanoparticles have been reported, which can deliver drugs and directly or indirectly activate the immune system to kill cancer cells (Hooftman and O'Neill, 2022; Luo et al., 2021; Sun et al., 2021). Although some nanomaterials have been used clinically in recent years, most of them have not achieved ideal clinical effects. In monotherapy, the continuing effects and biosafety of NPs on tumor cells require further study.

There is a new type of cell death called ferroptosis that differs from apoptosis, necrosis, and autophagy, which are all iron-dependent cell deaths (Chen et al., 2021b; Tang et al., 2021). As a metabolic disorder resulting from iron, ROS, and polyunsaturated fats, ferroptosis is characterized by deranged iron metabolism. Iron, lipid, and energy metabolism play a significant role in the sensitivity of tumor cells to ferroptosis (Lee et al., 2020; Li and Li, 2020; Jiang et al., 2021). Nanomedicine has become a new direction in the application of ferroptosis. Ultra-small PEG@ SiO_2_ NPs induce ferroptosis and limit tumor growth in starving cancer cells by mediating iron overuptake (Ma et al., 2017). In addition, p53 plasmid-coated metal-organic network NPs lead to ferroptosis and tumor growth inhibition by blocking GSH synthesis (Zheng et al., 2017). In our study, we found that PPy@Fe_3_O_4_ NPs restrained CRC cells’ growth and metastasis by promoting cell ferroptosis, and the exact mechanism needs to be further studied.

In normal physiology processes, NF-κB pathway coordinates the inflammatory process and participates in the regulation of various steps of the cell cycle and survival (DiDonato et al., 2012; Zhang et al., 2017). Binding to an inhibitory protein in the cytoplasm keeps it inactive. In response to the signal, its inhibitor is phosphorylated and proteolytically degraded, and NF-κB is translocated vigorously to the nucleus, where it promotes transcription of target genes (Vaiopoulos et al., 2013). Numerous pieces of evidence indicate that NF-κB has a key role in the initiation and propagation of CRC. Furthermore, the NF-κB signaling activation has been identified as a recognized event in the EMT process (Min et al., 2008). Liu et al. found that DCLK1 facilitates EMT via the NF-κB signaling pathway in CRC cells (Liu et al., 2018). Moreover, previous studies have shown that NF-κB regulates Vimentin and Snail expression directly by binding their promoters (Wu et al., 2004), which is consistent with our WB results. Herein, our study demonstrated that PPy@Fe_3_O_4_ NPs inhibit CRC cell proliferation and metastasis by blocking the NF-κB signaling pathway.

Overall, we exhibited the suppressive role of NPs in the progression of CRC *in vitro* and *in vivo*. Furthermore, our results revealed that PPy@Fe_3_O_4_ has an excellent photothermal effect and photostability under NIR irradiation. PPy@Fe_3_O_4_ NPs can not only be used for PPT and CDT but also can inhibit the growth and metastasis of tumor cells by regulating the NF-κB signaling pathway. Therefore, a therapeutic strategy based on PPy@Fe_3_O_4_ NPs to attenuate tumor development may be a potential approach for CRC treatment.

## Conclusion

PPy is a common non-toxic conductive polymer that is slightly soluble in water, other nanomaterials loaded with PPy can significantly improve their photothermal effect. In this study, we developed an NPs (PPy@Fe_3_O_4_) based on PPy to enhance the effect of PTT/CDT in CRC. The NPs displayed a high photothermal conversion efficiency of 52% because of PPy, which was much higher than that of traditional PPT agents. Besides, NPs were responsively decomposed in the tumor microenvironment to release the Fe ions of different valences, which promoted the generation of toxic OH from H_2_O_2_ for CDT. More importantly, we discovered a direct effect of NPs on colorectal cancer cells. PPy@Fe_3_O_4_ NPs can inhibit the growth and metastasis of colorectal cancer cells through the NF-κB signaling pathway, and promote cell ferroptosis.

## Data Availability

The original contributions presented in the study are included in the article/Supplementary Material, further inquiries can be directed to the corresponding authors.

## References

[B1] Aggarwal C. Meropol N. J. Punt C. J. Iannotti N. Saidman B. H. Sabbath K. D. (2013). Relationship among circulating tumor cells, CEA and overall survival in patients with metastatic colorectal cancer. Ann. Oncol. 24 (2), 420–428. 10.1093/annonc/mds336 23028040

[B2] Aksan A. Farrag K. Aksan S. Schroeder O. Stein J. (2021). Flipside of the coin: Iron deficiency and colorectal cancer. Front. Immunol. 12, 635899. 10.3389/fimmu.2021.635899 33777027 PMC7991591

[B3] Baek S. Singh R. K. Khanal D. Patel K. D. Lee E. J. Leong K. W. (2016). Smart multifunctional drug delivery towards anticancer therapy harmonized in mesoporous nanoparticles. Nanoscale 7 (34), 14191–14216. 10.1039/c5nr02730f 26260245

[B4] Biller L. H. Schrag D. (2021). Diagnosis and treatment of metastatic colorectal cancer: A review. JAMA 325 (7), 669–685. 10.1001/jama.2021.0106 33591350

[B5] Bray F. Ferlay J. Soerjomataram I. Siegel R. L. Torre L. A. Jemal A. (2018). Global cancer statistics 2018: GLOBOCAN estimates of incidence and mortality worldwide for 36 cancers in 185 countries. CA A Cancer J. Clin. 68 (6), 394–424. 10.3322/caac.21492 30207593

[B6] Brenner H. Kloor M. Pox C. P. (2014). Colorectal cancer. Lancet 383 (9927), 1490–1502. 10.1016/S0140-6736(13)61649-9 24225001

[B7] Cen D. Ge Q. Xie C. Zheng Q. Guo J. Zhang Y. (2021). ZnS@BSA nanoclusters potentiate efficacy of cancer immunotherapy. Adv. Mat. 33 (49), e2104037. 10.1002/adma.202104037 34622500

[B8] Chen P. Li X. Zhang R. Liu S. Xiang Y. Zhang M. (2020). Combinative treatment of β-elemene and cetuximab is sensitive to KRAS mutant colorectal cancer cells by inducing ferroptosis and inhibiting epithelial-mesenchymal transformation. Theranostics 10 (11), 5107–5119. 10.7150/thno.44705 32308771 PMC7163451

[B9] Chen X. Kang R. Kroemer G. Tang D. (2021b). Broadening horizons: The role of ferroptosis in cancer. Nat. Rev. Clin. Oncol. 18 (5), 280–296. 10.1038/s41571-020-00462-0 33514910

[B10] Chen X. Li J. Kang R. Klionsky D. J. Tang D. (2021a). Ferroptosis: Machinery and regulation. Autophagy 17 (9), 2054–2081. 10.1080/15548627.2020.1810918 32804006 PMC8496712

[B11] DiDonato J. A. Mercurio F. Karin M. (2012). NF-κB and the link between inflammation and cancer. Immunol. Rev. 246 (1), 379–400. 10.1111/j.1600-065X.2012.01099.x 22435567

[B12] Duan X. Chan C. Lin W. (2019). Nanoparticle-Mediated immunogenic cell death enables and potentiates cancer immunotherapy. Angew. Chem. Int. Ed. 58 (3), 670–680. 10.1002/anie.201804882 PMC7837455 30016571

[B13] El-Toni A. M. Habila M. A. Labis J. P. ALOthman Z. A. Alhoshan M. Elzatahry A. A. (2016). Design, synthesis and applications of core-shell, hollow core, and nanorattle multifunctional nanostructures. Nanoscale 8 (5), 2510–2531. 10.1039/c5nr07004j 26766598

[B14] Engstrand J. Nilsson H. Strömberg C. Jonas E. Freedman J. (2018). Colorectal cancer liver metastases - a population-based study on incidence, management and survival. BMC Cancer 18 (1), 78. 10.1186/s12885-017-3925-x 29334918 PMC5769309

[B15] Enriquez-Navas P. M. Wojtkowiak J. W. Gatenby R. A. (2015). Application of evolutionary principles to cancer therapy. Cancer Res. 75 (22), 4675–4680. 10.1158/0008-5472.can-15-1337 26527288 PMC4693617

[B16] Fan A. Wang B. Wang X. Nie Y. Fan D. Zhao X. (2021). Immunotherapy in colorectal cancer: Current achievements and future perspective. Int. J. Biol. Sci. 17 (14), 3837–3849. 10.7150/ijbs.64077 34671202 PMC8495390

[B17] Guan D. Zhou W. Wei H. Wang T. Zheng K. Yang C. (2022c). Ferritinophagy-Mediated ferroptosis and activation of keap1/nrf2/HO-1 pathway were conducive to EMT inhibition of gastric cancer cells in action of 2,2'-Di-pyridineketone hydrazone dithiocarbamate butyric acid ester. Oxid. Med. Cell. Longev. 2022, 3920664. 10.1155/2022/3920664 35237380 PMC8885181

[B18] Guan S. Liu X. Fu Y. Li C. Wang J. Mei Q. (2022a). A biodegradable "Nano-donut" for magnetic resonance imaging and enhanced chemo/photothermal/chemodynamic therapy through responsive catalysis in tumor microenvironment. J. Colloid Interface Sci. 608 (1), 344–354. 10.1016/j.jcis.2021.09.186 34626980

[B19] Guan S. Liu X. Li C. Wang X. Cao D. Wang J. (2022b). Intracellular mutual amplification of oxidative stress and inhibition multidrug resistance for enhanced sonodynamic/chemodynamic/chemo therapy. Small 18 (13), e2107160. 10.1002/smll.202107160 35146899

[B20] Hooftman A. O'Neill L. A. J. (2022). Nanoparticle asymmetry shapes an immune response. Nature 601 (7893), 323–325. 10.1038/d41586-021-03806-7 35046597

[B21] Huang G. Ma L. Shen L. Lei Y. Guo L. Deng Y. (2022). MIF/SCL3A2 depletion inhibits the proliferation and metastasis of colorectal cancer cells via the AKT/GSK-3β pathway and cell iron death. J. Cell. Mol. Med. 26 (12), 3410–3422. 10.1111/jcmm.17352 35567291 PMC9189354

[B22] Huang X. Deng G. Han Y. Yang G. Zou R. Zhang Z. (2019). Right Cu _2−_ x S@MnS core–shell nanoparticles as a photo/H _2_ O _2_ -responsive platform for effective cancer theranostics. Adv. Sci. (Weinh). 6 (20), 1901461. 10.1002/advs.201901461 31637173 PMC6794717

[B23] Jiang W. G. Sanders A. J. Katoh M. Ungefroren H. Gieseler F. Prince M. (2015). Tissue invasion and metastasis: Molecular, biological and clinical perspectives. Semin. Cancer Biol. 35, S244–S275. 10.1016/j.semcancer.2015.03.008 25865774

[B24] Jiang X. Stockwell B. R. Conrad M. (2021). Ferroptosis: Mechanisms, biology and role in disease. Nat. Rev. Mol. Cell Biol. 22 (4), 266–282. 10.1038/s41580-020-00324-8 33495651 PMC8142022

[B25] Koppe M. J. Bleichrodt R. P. Oyen W. J. Boerman O. C. (2005). Radioimmunotherapy and colorectal cancer. Br. J. Surg. 92 (3), 264–276. 10.1002/bjs.4936 15739250

[B26] Lee H. Zandkarimi F. Zhang Y. Meena J. K. Kim J. Zhuang L. (2020). Energy-stress-mediated AMPK activation inhibits ferroptosis. Nat. Cell Biol. 22 (2), 225–234. 10.1038/s41556-020-0461-8 32029897 PMC7008777

[B27] Li D. Li Y. (2020). The interaction between ferroptosis and lipid metabolism in cancer. Signal Transduct. Target. Ther. 5 (1), 108. 10.1038/s41392-020-00216-5 32606298 PMC7327075

[B28] Li X. Li W. Wang M. Liao Z. (2021). Magnetic nanoparticles for cancer theranostics: Advances and prospects. J. Control. Release 335, 437–448. 10.1016/j.jconrel.2021.05.042 34081996

[B29] Liang Y. Mitriashkin A. Lim T. T. Goh J. C. (2021). Conductive polypyrrole-encapsulated silk fibroin fibers for cardiac tissue engineering. Biomaterials 276, 121008. 10.1016/j.biomaterials.2021.121008 34265591

[B30] Liu W. Wang S. Sun Q. Yang Z. Liu M. Tang H. (2018). *Retracted:* DCLK1 promotes epithelial-mesenchymal transition via the PI3K/akt/NF-κB pathway in colorectal cancer. Int. J. Cancer 142 (10), 2068–2079. 10.1002/ijc.31232 29277893

[B31] Luo L. Li X. Zhang J. Zhu C. Jiang M. Luo Z. (2021). Enhanced immune memory through a constant photothermal-metabolism regulation for cancer prevention and treatment. Biomaterials 270, 120678. 10.1016/j.biomaterials.2021.120678 33517205

[B32] Ma P. Xiao H. Yu C. Liu J. Cheng Z. Song H. (2017). Enhanced cisplatin chemotherapy by iron oxide nanocarrier-mediated generation of highly toxic reactive oxygen species. Nano Lett. 17 (2), 928–937. 10.1021/acs.nanolett.6b04269 28139118

[B33] Miar S. Ong J. L. Bizios R. Guda T. (2021). Electrically stimulated tunable drug delivery from polypyrrole-coated polyvinylidene fluoride. Front. Chem. 9, 599631. 10.3389/fchem.2021.599631 33614599 PMC7892451

[B34] Min C. Eddy S. F. Sherr D. H. Sonenshein G. E. (2008). NF-κB and epithelial to mesenchymal transition of cancer. J. Cell. Biochem. 104, 733–744. 10.1002/jcb.21695 18253935

[B35] Modest D. P. Pant S. Sartore-Bianchi A. (2019). Treatment sequencing in metastatic colorectal cancer. Eur. J. Cancer 109, 70–83. 10.1016/j.ejca.2018.12.019 30690295

[B36] Morgan M. J. Liu Z. G. (2011). Crosstalk of reactive oxygen species and NF-κB signaling. Cell Res. 21 (1), 103–115. 10.1038/cr.2010.178 21187859 PMC3193400

[B37] Padmanabhan H. Brookes M. J. Iqbal T. (2015). Iron and colorectal cancer: Evidence from *in vitro* and animal studies. Nutr. Rev. 73 (5), 308–317. 10.1093/nutrit/nuu015 26011904

[B38] Patel M. Horgan P. G. McMillan D. C. Edwards J. (2018). NF-κB pathways in the development and progression of colorectal cancer. Transl. Res. 197, 43–56. 10.1016/j.trsl.2018.02.002 29550444

[B39] Phipps O. Brookes M. J. Al-Hassi H. O. (2021). Iron deficiency, immunology, and colorectal cancer. Nutr. Rev. 79 (1), 88–97. 10.1093/nutrit/nuaa040 32679587

[B40] Ploug M. Kroijer R. Qvist N. Lindahl C. H. Knudsen T. (2021). Iron deficiency in colorectal cancer patients: A cohort study on prevalence and associations. Colorectal Dis. 23 (4), 853–859. 10.1111/codi.15467 33253490

[B41] Rebersek M. (2020). Consensus molecular subtypes (CMS) in metastatic colorectal cancer -personalized medicine decision. Radiol. Oncol. 54 (3), 272–277. 10.2478/raon-2020-0031 32463385 PMC7409603

[B42] Setyawati M. I. Leong D. T. (2017). Mesoporous silica nanoparticles as an antitumoral-angiogenesis strategy. ACS Appl. Mat. Interfaces 9 (8), 6690–6703. 10.1021/acsami.6b12524 28150492

[B43] Siegel R. L. Miller K. D. Fuchs H. E. Jemal A. (2021). Cancer statistics, 2021. Ca. A Cancer J. Clin. 71 (1), 7–33. 10.3322/caac.21654 33433946

[B44] Su L. J. Zhang J. H. Gomez H. Murugan R. Hong X. Xu D. (2019). Reactive oxygen species-induced lipid peroxidation in apoptosis, autophagy, and ferroptosis. Oxid. Med. Cell. Longev. 2019, 1–13. 10.1155/2019/5080843 PMC6815535 31737171

[B45] Sun T. Zhang Y. S. Pang B. Hyun D. C. Yang M. Xia Y. (2014). Engineered nanoparticles for drug delivery in cancer therapy. Angew. Chem. Int. Ed. Engl. 53 (46), 12320–12364. 10.1002/anie.201403036 25294565

[B46] Sun W. Ge K. Jin Y. Han Y. Zhang H. Zhou G. (2019). Bone-targeted nanoplatform combining zoledronate and photothermal therapy to treat breast cancer bone metastasis. ACS Nano 13 (7), 7556–7567. 10.1021/acsnano.9b00097 31259530

[B47] Sun X. Zhang Y. Li J. Park K. S. Han K. Zhou X. (2021). Amplifying STING activation by cyclic dinucleotide-manganese particles for local and systemic cancer metalloimmunotherapy. Nat. Nanotechnol. 16 (11), 1260–1270. 10.1038/s41565-021-00962-9 34594005 PMC8595610

[B48] Tang D. Chen X. Kang R. Kroemer G. (2021). Ferroptosis: Molecular mechanisms and health implications. Cell Res. 31 (2), 107–125. 10.1038/s41422-020-00441-1 33268902 PMC8026611

[B49] Tang Z. Liu Y. He M. Bu W. (2019). Chemodynamic therapy: Tumour microenvironment-mediated Fenton and fenton-like reactions. Angew. Chem. Int. Ed. 58 (4), 946–956. 10.1002/anie.201805664 30048028

[B50] Torti S. V. Manz D. H. Paul B. T. Blanchette-Farra N. Torti F. M. (2018). Iron and cancer. Annu. Rev. Nutr. 38, 97–125. 10.1146/annurev-nutr-082117-051732 30130469 PMC8118195

[B51] Torti S. V. Torti F. M. (2020). Iron and cancer: 2020 vision. Cancer Res. 80 (24), 5435–5448. 10.1158/0008-5472.CAN-20-2017 32928919 PMC8118237

[B52] Vaiopoulos A. G. Athanasoula K. C. Papavassiliou A. G. (2013). NF-κB in colorectal cancer. J. Mol. Med. 91 (9), 1029–1037. 10.1007/s00109-013-1045-x 23636511

[B53] Wilson M. J. Harlaar J. J. Jeekel J. Schipperus M. Zwaginga J. J. (2018). Iron therapy as treatment of anemia: A potentially detrimental and hazardous strategy in colorectal cancer patients. Med. Hypotheses 110, 110–113. 10.1016/j.mehy.2017.12.011 29317052

[B54] Wu Y. Diab I. Zhang X. Izmailova E. S. Zehner Z. E. (2004). Stat3 enhances vimentin gene expression by binding to the antisilencer element and interacting with the repressor protein, ZBP-89. Oncogene 23, 168–178. 10.1038/sj.onc.1207003 14712222

[B55] Xu J. Fan J. Qin X. Cai J. Gu J. Wang S. (2019). Chinese guidelines for the diagnosis and comprehensive treatment of colorectal liver metastases (version 2018). J. Cancer Res. Clin. Oncol. 145 (3), 725–736. 10.1007/s00432-018-2795-1 30542791 PMC11810244

[B56] Yu N. Qiu P. Ren Q. Wen M. Geng P. Macharia D. K. (2021). Transforming a sword into a knife: Persistent phototoxicity inhibition and alternative therapeutical activation of highly-photosensitive phytochlorin. ACS Nano 15 (12), 19793–19805. 10.1021/acsnano.1c07241 34851096

[B57] Zhang L. Forgham H. Shen A. Qiao R. Guo B. (2022). Recent advances in single Fe-based nanoagents for photothermal-chemodynamic cancer therapy. Biosens. (Basel) 12 (2), 86. 10.3390/bios12020086 PMC8869282 35200346

[B58] Zhang Q. Lenardo M. J. Baltimore D. (2017). 30 Years of NF-κB: A blossoming of relevance to human pathobiology. Cell 168 (1-2), 37–57. 10.1016/j.cell.2016.12.012 28086098 PMC5268070

[B59] Zheng D. W. Lei Q. Zhu J. Y. Fan J. X. Li C. X. Li C. (2017). Switching apoptosis to ferroptosis: Metal-organic network for high-efficiency anticancer therapy. Nano Lett. 17 (1), 284–291. 10.1021/acs.nanolett.6b04060 28027643

[B60] Zheng N. Wang Q. Li C. Wang X. Liu X. Wang X. (2021). Responsive degradable theranostic agents enable controlled selenium delivery to enhance photothermal radiotherapy and reduce side effects. Adv. Healthc. Mat. 10 (10), e2002024. 10.1002/adhm.202002024 33645002

[B61] Zheng N. N. Kong W. Y. Huang Z. Liu X. J. Liang S. H. Deng G. Y. (2022). Novel theranostic nanoagent based on CuMo2S3-PEG-Gd for MRI-guided photothermal/photodynamic/chemodynamic therapy. Rare Met. 41 (1), 45–55. 10.1007/s12598-021-01793-2

[B62] Zhou X. Yang A. Huang Z. Yin G. Pu X. Jin J. (2017). Enhancement of neurite adhesion, alignment and elongation on conductive polypyrrole-poly (lactide acid) fibers with cell-derived extracellular matrix. Colloids Surf. B Biointerfaces 149, 217–225. 10.1016/j.colsurfb.2016.10.014 27768911

[B63] Zhu X. Feng W. Chang J. Tan Y. W. Li J. Chen M. (2016). Temperature-feedback upconversion nanocomposite for accurate photothermal therapy at facile temperature. Nat. Commun. 7, 10437. 10.1038/ncomms10437 26842674 PMC4742858

