# Corrigendum: Yu et al. PPy@Fe_3_O_4_ nanoparticles inhibit the proliferation and metastasis of CRC *via* suppressing the NF-κB signaling pathway and promoting ferroptosis

**DOI:** 10.3389/fbioe.2022.1094064

**Published:** 2022-12-01

**Authors:** Zhilong Yu, Shanshi Tong, Chenyi Wang, Zizhen Wu, Yingjiang Ye, Shan Wang, Kewei Jiang

**Affiliations:** ^1^ Department of Gastroenterological Surgery, Laboratory of Surgical Oncology, Beijing Key Laboratory of Colorectal Cancer Diagnosis and Treatment Research, Peking University People’s Hospital, Beijing, China; ^2^ State Key Laboratory of Oncogenes and Related Genes, Shanghai Cancer Institute, Renji Hospital, School of Medicine, Shanghai Jiao Tong University, Shanghai, China

**Keywords:** colorectal cancer, nanoparticles, metastasis, NF-κB, ferroptosis

In the published article, there was an error in the legend for Figure 6 as published. “DLD1” in Figure 6B Legend was incorrectly written as “MGC-803”. The corrected legend appears below.

In the published article, there was an error in Figure 5C and Figure 6B as published. “Invasion” was incorrectly written as “Invation”. The real image in the lower right corner of Figure 5C was incorrect due to a copy error when assembling the images. “NIR” in Figure 6B was incorrectly written as “H_2_O_2_”. “Control” in Figure 6F was incorrectly written as “Contol”. The corrected Figure 5 and Figure 6 and its caption appear below.

**FIGURE 5:**
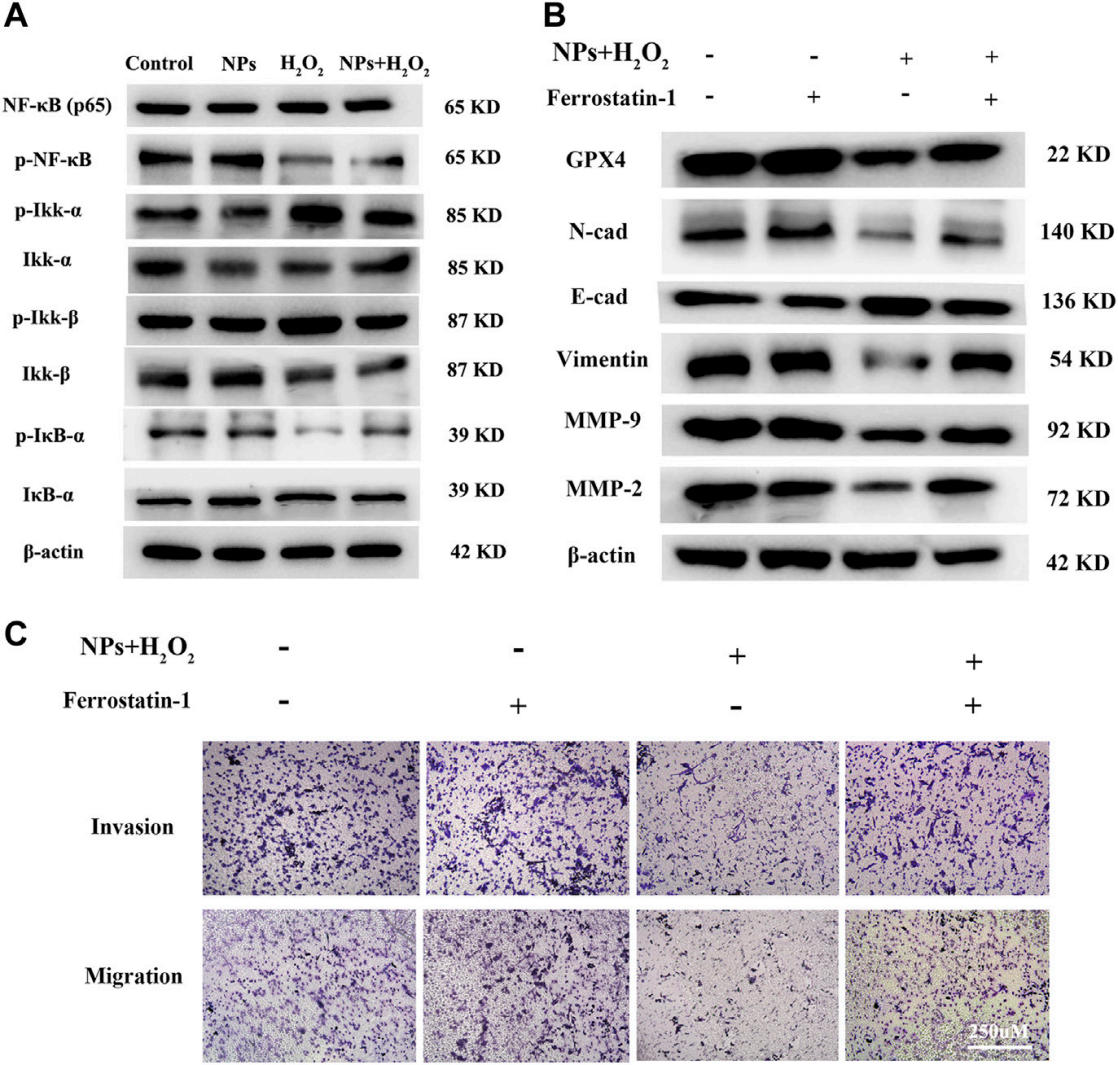
PPy@ Fe_3_O_4_ NPs suppress CRC cells metastasis by promoting cell ferroptosis and inhibiting NF-κB signaling pathway. **(A)** Western blot. Colorectal cancer cell line DLD1 was treated with various groups (Control, H_2_O_2_, NPs and NPs +H_2_O_2_), and then subjected to Western blot analysis of the key proteins of the NF-κB signaling pathway (Ikk-β, p-Ikk-β, Ikk-α, p-Ikk-α, NF-κB, p-NF-κB, iκB-α, and p-iκB-α). **(B)** Effects of the ferroptosis inhibitor Ferrostatin-1 on PPy@Fe_3_O_4_ NPs-induced metastasis-related proteins expression. **(C)** Transwell showed that PPy@Fe_3_O_4_ NPs induced cell migration and invasion were abolished after addition of the ferroptosis inhibitor Ferrostatin-1 in CRC cells.

**FIGURE 6:**
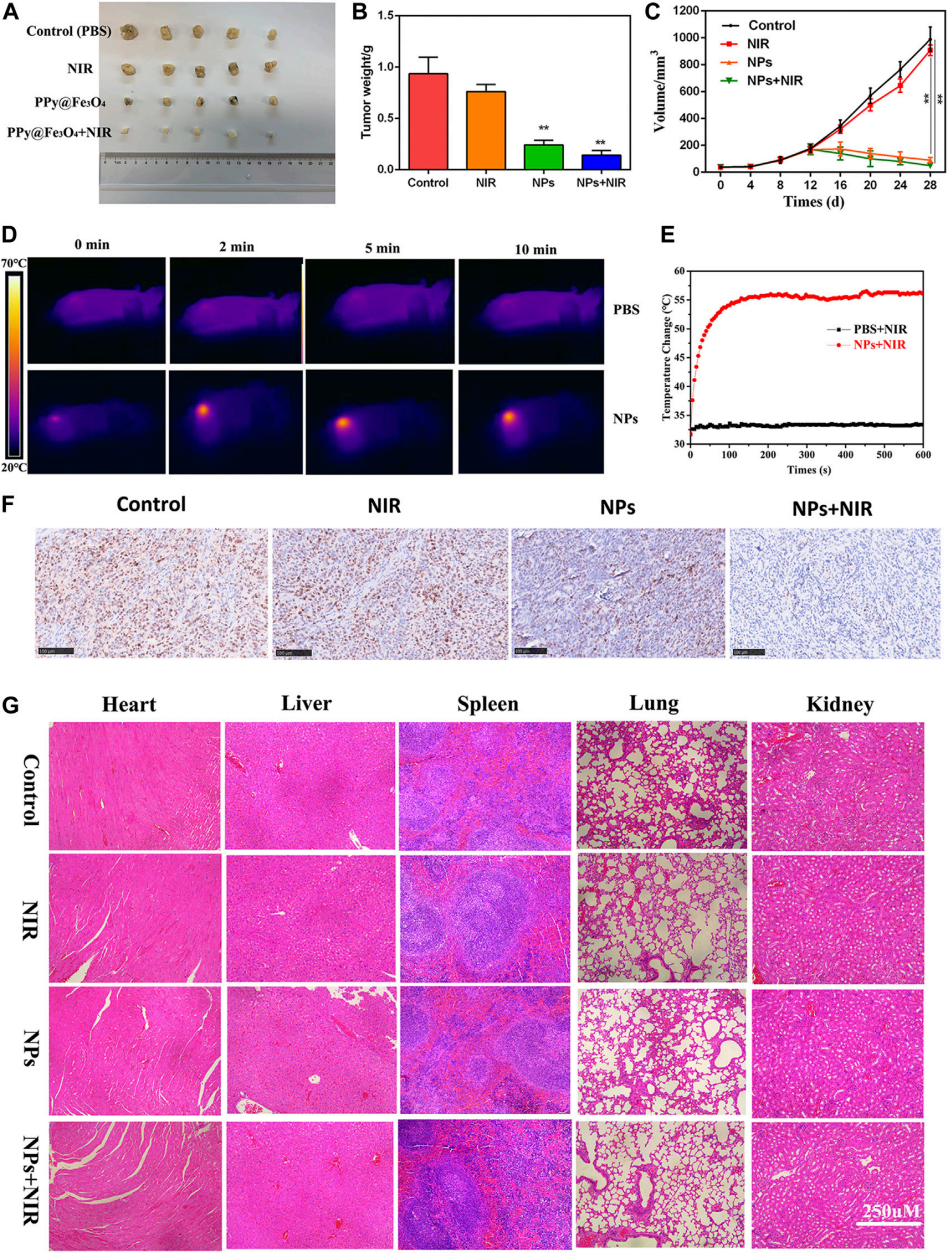
Anti-tumour activity of PPy@ Fe_3_O_4_ NPs in nude mouse tumour cell xenografts. **(A)** Images of subcutaneous xenograft tumors of DLD1 cells. **(B)** The final tumor weight of DLD1 cells was shown. **(C)** The tumor volume and change of different groups. **(D)** The temperature change and **(E)** infrared thermal imaging of the mice injected with PBS, NPs under laser irradiation. **(F)** Ki67 staining of the tumors in the control group and other treated groups. (scale bar: 100 μm). **(G)** H&E staining of the main organs from the control and treatment groups. (scale bar: 250 μm). **p < 0.01.

The authors apologize for this error and state that this does not change the scientific conclusions of the article in any way. The original article has been updated.

