# Corrigendum: PPy@Fe_3_O_4_ nanoparticles inhibit the proliferation and metastasis of CRC via suppressing the NF-κB signaling pathway and promoting ferroptosis

**DOI:** 10.3389/fbioe.2023.1148674

**Published:** 2023-09-27

**Authors:** Zhilong Yu, Shanshi Tong, Chenyi Wang, Zizhen Wu, Yingjiang Ye, Shan Wang, Kewei Jiang

**Affiliations:** ^1^ Department of Gastroenterological Surgery, Laboratory of Surgical Oncology, Beijing Key Laboratory of Colorectal Cancer Diagnosis and Treatment Research, Peking University People’s Hospital, Beijing, China; ^2^ State Key Laboratory of Oncogenes and Related Genes, Shanghai Cancer Institute, Renji Hospital, School of Medicine, Shanghai Jiao Tong University, Shanghai, China

**Keywords:** colorectal cancer, nanoparticles, metastasis, NF-κB, ferroptosis

In the published article, there was an error in Figures 2E, 5 as published. Modifications to UV absorption spectra in Figure 2E and NF-κB related protein typographical errors in the WB experiment in Figure 5 were made after a recheck of the figures, but not included in the final article. The corrected Figures 2, 5 and their captions appear below.

**FIGURE 2:**
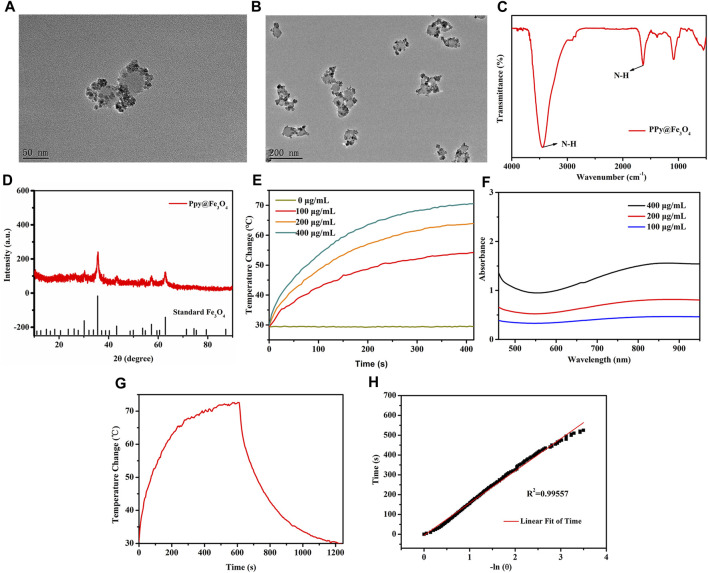
The characterization and photothermal properties of the PPy@Fe_3_O_4_ NPs. **(A,B)** High and Low TEM images of PPy@Fe_3_O_4_ NPs; **(C)** FTIR spectra of PPy@Fe_3_O_4_ NPs; **(D)** XRD spectra of PPy@Fe_3_O_4_ NPs; **(E)** UV-Vis-NIR absorption spectra of PPy@Fe_3_O_4_ NPs at different concentrations; **(F)** Temperature change curve with various concentrations of NPs; **(G)** Temperature curve of rising with irradiation and naturally cooling; **(H)** Linear regression curve of cooling process (red).

**FIGURE 5:**
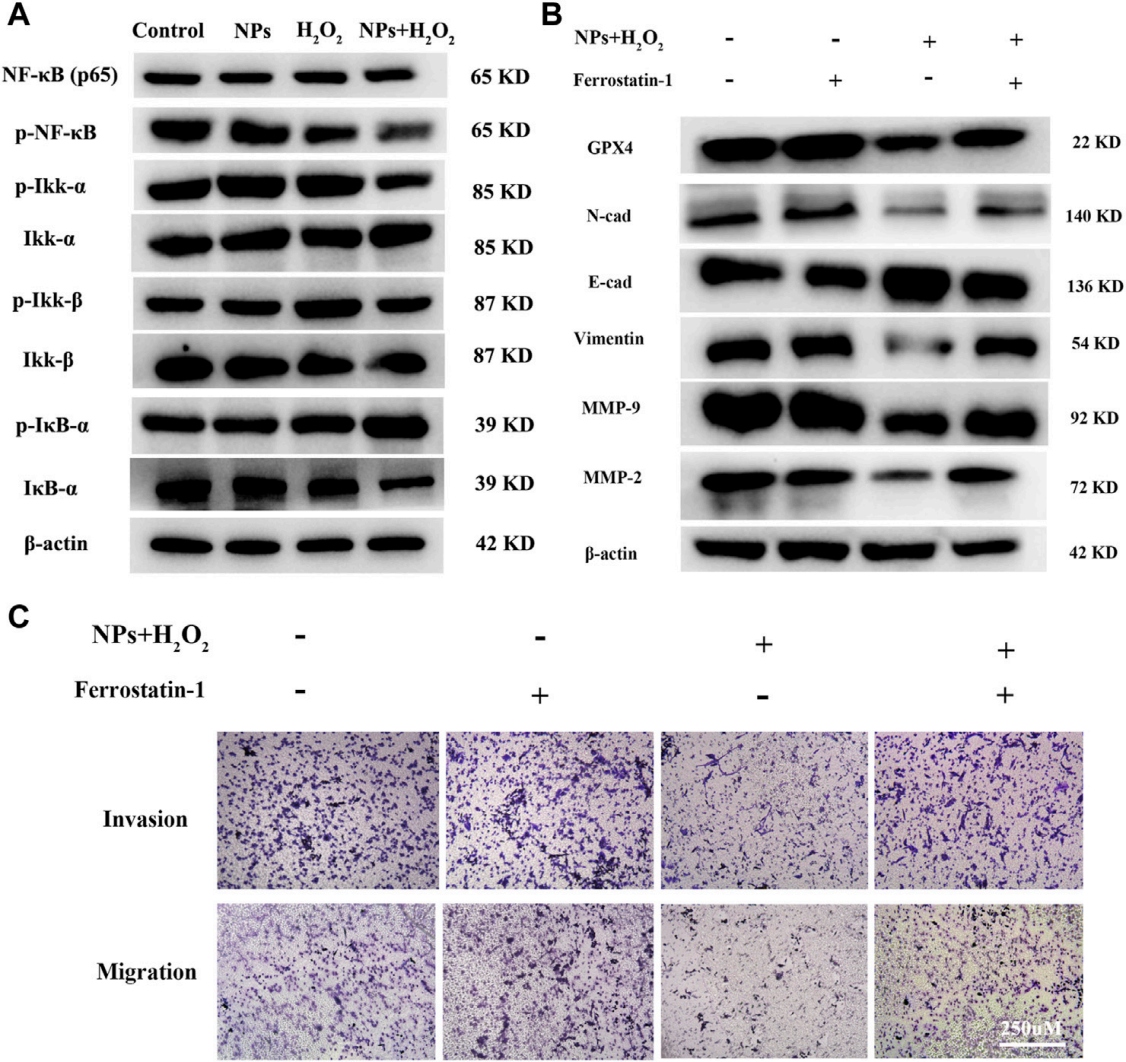
PPy@Fe_3_O_4_ NPs suppress CRC cells metastasis by promoting cell ferroptosis and inhibiting NF-κB signaling pathway. **(A)** Western blot. Colorectal cancer cell line DLD1 was treated with various groups (Control, H_2_O_2_, NPs and NPs + H_2_O_2_), and then subjected to Western blot analysis of the key proteins of the NF-KB signaling pathway (Ikk-β, p-Ikk-β, ikk-α, p-Ikk-α, NF-κβ, p-NF-κβ, IκB-α and p-IκB-α). **(B)** Effects of the ferroptosis inhibitor Ferrostatin-1 on PPy@Fe_3_O_4_ NPs-induced metastasis-related proteins expression. **(C)** Transwell showed that PPy@Fe_3_O_4_ NPs-induced cell migration and invasion were abolished after addition of the ferroptosis inhibitor Ferrostatin-1 in CRC cell.

Additionally, the statistical method in the “Statistics” section and “PPy@Fe3O4 NPs inhibited EMT via the NF-κB signaling pathway” section were incorrect due to translation error and clerical error.

A correction has been made to **Materials and methods** section, subsection *Statistics*. A correction has also been made to the **Results** section, subsection *PPy@Fe3O4 NPs inhibited EMT via the NF-κB signaling pathway*, Paragraph 1. These sentences previously stated, respectively:

“Based on experiments performed in triplicate for cell proliferation, migration, and invasion, all data are presented as mean ± SEM. In the animal study of nude mice, the data are presented as mean ± SEM of 5 mice. Statistical analyses were performed with the χ2 test or the Student’s t-test (two-tailed unpaired). All the data were analyzed using SPSS 22.0 software and *p* < 0.05 was considered significant.”

“There was an increase in the levels of IKKα and IKKβ in DLD1, as well as a decrease in the amounts of IκBα after treatment with H_2_O_2_. P65 levels did not change significantly, but phospho-p65 expression increased. We discovered that the expression of phosphorylated (p)p65, IKKα, IKKβ, and IκBα, which are essential for activating the NF-κB signaling pathway, were downregulated by NPs with H_2_O in DLD1 cells.”

The corrected sentences appear below:

“All data are presented as mean ± SD. Statistical analyses were performed with the χ2 test or the Student’s t-test (two-tailed unpaired). All the data were analyzed using Origin and Graphpad. Moreover, *p* < 0.05 is considered statistically significant.”

“There was a decrease in the levels of p-IKKα and p-IKKβ in DLD1, as well as an increase in the amounts of p-IκBα after treatment with NPs and H_2_O_2_. P65 levels did not change significantly, but phospho-p65 expression decreased. We discovered that the expression of phosphorylated (p)p65, p-IKKα, p-IKKβ, and IκBα, which are essential for activating the NF-κB signaling pathway, were downregulated by NPs with H_2_O_2_ in DLD1 cells.”

There was also an error in the **Funding** statement. National Nature Science Foundation of China (No. 81871962) has expired and ceased. The correct **Funding** statement appears below.

“This study was supported by the National Scientific Center Project (No. 62088101) and the Industry-University-Research Innovation Fund in Ministry of Education of the People’s Republic of China (No. 2018A01013).”

The authors apologize for these errors and state that this does not change the scientific conclusions of the article in any way. The original article has been updated.

